# Do Americans Need More Calcium?

**Published:** 1919-12

**Authors:** 


### DO AMERICANS NEED MORE CALCIUM?

Professor Sherman of Columbia University, who has devoted much attention to the inorganic metabolism of man, has come to the conclusion that "the ordinary mixed diet of Americans and Europeans, at least among dwellers in cities and towns, is probably more often deficient in calcium than in any other chemical element." This statement is rendered additionally significant by the fact that calcium constitutes about 2 per cent. of the body weight, a proportion larger than that of any other of the inorganic elements found in the organism. In the case of the growing individual, lime must be provided for the normal development of the bones, which include 99 per cent. of this element. The pregnant mother needs calcium for the rapid increment of the fetus, and during lactation she has a similar requirement for the production of a secretion, milk, which is exceptionally rich in it. The tendency toward a shortage of calcium is not confined to the human species. Forbes, of the Ohio Experiment Station, has summarized the available information to show that liberal milk production in cattle involves a certain degree of impoverishment of the skeleton in mineral substance; that rations which contain no leguminous roughage are apt to be definitely lacking in mineral nutriment, especially calcium; that the response of heavily producing cows to liberal intake of mineral nutriment is remarkable for its efficiency, it being apparently impossible by any method of feeding entirely to prevent loss of calcium, at least during the early part of the period of lactation, and that the mineral constituents of the skeleton appear to be more readily available for use in milk secretion than are nutriments directly absorbed from the ration.

The seeds, such as are represented by the numerous cereals and their milling products that enter largely into our present day diet, the roots and tubers, and the various meat products probably constitute more than three quarters of the food intake of the average adult American. All of these food materials are comparatively poor in calcium. In referring to this deficiency, McCollum, Simmonds and Parsons of the School of Hygiene at Johns Hopkins University, regard it as so pronounced that they are of the opinion that "even in those human dietaries in which such calcium-rich foods as milk are used in fair liberality, the intake of calcium may be still below the optimum, and that a direct addition of this element in the form of the carbonate or lactate might be of distinct benefit in human nutrition except perhaps in those regions where the water is unusually rich in calcium salts."

Oscar Loew long since suggested the addition of calcium salts to the dough from which bread is baked. During the war, several European investigators have formulated similar advice in the belief that even when the daily losses of lime are inconspicuous, long continued deficits in the human calcium balance may be a disadvantage. The average daily calcium requirement has been estimated as a little less than 0.5 gm. per man. One pint of milk will furnish this quantity, but aside from this food, the menu of American homes offers relatively few opportunities to satisfy the human need. Admitting, as we now freely may, that the calcium requirement can be satisfied directly by inorganic salts, it remains to be determined whether the unfortunate poverty of American dietaries in milk should not be artificially compensated for. McCollum and his collaborators remind up that since civilized man usually adds sodium chlorid to his foods to suit the taste, the shortage of sodium and chlorin in the diet of man presents no problem. They propose that an addition of calcium could be most conveniently made to our foods through the use of a mixture of equal parts of common salt and of calcium carbonate in the kitchen and on the table. This suggestion deserves a sympathetic hearing.—*Journal A. M. A.* September 20, 1919.

